# Polymorphisms of Antioxidant Enzymes SOD2 (rs4880) and GPX1 (rs1050450) Are Associated with Bladder Cancer Risk or Its Aggressiveness

**DOI:** 10.3390/medicina59010131

**Published:** 2023-01-09

**Authors:** Predrag Nikic, Dejan Dragicevic, Djurdja Jerotic, Slaviša Savic, Tatjana Djukic, Branko Stankovic, Luka Kovacevic, Tatjana Simic, Marija Matic

**Affiliations:** 1 Clinic of Urology, Clinical Center of Serbia, 11000 Belgrade, Serbia; 2 Faculty of Medicine, University of Belgrade, 11000 Belgrade, Serbia; 3 Institute of Medical and Clinical Biochemistry, 11000 Belgrade, Serbia; 4 Department of Urology, KBC Dr. Dragiša Mišovic, 11000 Belgrade, Serbia; 5 Serbian Academy of Sciences and Arts, 11000 Belgrade, Serbia

**Keywords:** urothelial bladder cancer, risk, genetic polymorphisms, SNP, GPX1, SOD2

## Abstract

*Background and Objectives*: Oxidative stress induced by increased reactive oxygen species (ROS) production plays an important role in carcinogenesis. The entire urinary tract is continuously exposed to numerous potentially mutagenic environmental agents which generate ROS during their biotransformation. In first line defense against free radicals, antioxidant enzymes superoxide dismutase (SOD2) and glutathione peroxidase (GPX1) both have essential roles. Altered enzyme activity and decreased ability of neutralizing free oxygen radicals as a consequence of genetic polymorphisms in genes encoding these two enzymes are well described so far. This study aimed to investigate the association of *GPX1 (rs1050450)* and *SOD2 (rs4880)* genetic variants with the urothelial bladder cancer (UBC) risk independently and in combination with smoking. Furthermore, we aimed to determine whether the UBC stage and pathological grade were influenced by *GPX1* and *SOD2* polymorphisms. *Material and Methods*: The study population included 330 patients with UBC (mean age 65 ± 10.3 years) and 227 respective controls (mean age 63.4 ± 7.9 years). Single nucleotide polymorphism (SNP) of *GPX1 (rs1050450)* was analyzed using the PCR-RFLP, while *SOD2 (rs4880)* SNP was analyzed using the q-PCR method. *Results:* Our results showed that UBC risk was significantly increased among carriers of at least one variant *SOD2 Val* allele compared to the *SOD2 Ala16Ala* homozygotes (OR = 1.55, *p* = 0.03). Moreover, this risk was even more pronounced in smokers with at least one variant *SOD2 Val* allele, since they have even 7.5 fold higher UBC risk (OR = 7.5, *p* < 0.001). Considering *GPX1* polymorphism, we have not found an association with UBC risk. However, *GPX1* genotypes distribution differed significantly according to the tumor stage (*p* ˂ 0.049) and pathohistological grade (*p* ˂ 0.018). *Conclusion:* We found that *SOD2* genetic polymorphism is associated with the risk of UBC development independently and in combination with cigarette smoking. Furthermore, we showed that *GPX1* genetic polymorphism is associated with the aggressiveness of the disease.

## 1. Introduction

Bladder cancer is the second most common genitourinary malignancy and the 10th most common cancer diagnosed worldwide [1]. In the developed world, the most common histological type of bladder cancer is urothelial carcinoma (UBC), with prominent male predominance, both in incidence and mortality rates [1,2]. This carcinoma is characterized by a significantly high risk for recurrence and progression, as well as suboptimal response to the existing systemic therapy. At the moment of diagnosis, 70% of UBC patients have non-muscle invasive bladder cancer (NMIBC) and during the natural history of the treated disease, 15–20% of them will progress to muscle-invasive bladder cancer (MIBC). The remaining 30% of UBC patients are primarily diagnosed with MIBC, as locally advanced or metastatic disease. After cystectomy, almost 50% of patients will relapse with local recurrence or more often with distant metastases [3]. Therefore, as a serious and potentially fatal disease, UBC requires lifelong monitoring and represents a large socio-economic burden. The entire urinary tract is continuously exposed to numerous potentially mutagenic environmental agents which are excreted in the urine. Chemicals found in tobacco and occupational chemicals represent the main risk factors of UBC [4,5,6]. It is widely accepted that chronic exposure to tobacco smoke and/or occupational carcinogens generates a production of reactive oxygen species (ROS) which induce genotoxic effects through CYP1A1-mediated metabolites and oxidative stress pathways [7]. A large number of studies have shown that oxidative stress plays an important role in carcinogenesis [8,9,10,11,12]. Namely, long-term accumulation of ROS leads to damage of all types of cellular and extracellular macromolecules, including DNA [13,14]. Additionally, increased production of ROS is assumed to affect the regulation of signaling pathways involved in cell proliferation, growth, survival, and apoptosis [15]. Moreover, ROS overproduction determines the maintenance of the inflammatory microenvironment which is favorable to carcinogenesis [12,14,16,17]. It was shown that high ROS levels may stimulate the production of proinflammatory cytokines and proangiogenic factors such as TNF-α, VEGF, and MMP-9, which are all linked with the carcinogenesis and tumor progression [18]. Additionally, a high ROS level may also induce NF-κB (nuclear factor kappa-light-chain-enhancer of activated B cells) and therefore modulate transcription of proinflammatory cytokines, including interleukins 6 and 8 (IL-6 and IL-8) [19,20]. 

In first-line defense against free radicals, antioxidant enzymes superoxide dismutase (SOD2) and glutathione peroxidase (GPX1) both have an essential roles. SOD2 converts ROS superoxide anion to hydrogen peroxide (H_2_O_2_), which is further reduced into the water by GPX1 activity. Altered enzyme activity and decreased ability to neutralize free oxygen radicals as a consequence of genetic polymorphisms in genes encoding these two enzymes are well described so far [21]. The single nucleotide polymorphism (SNP) in the *SOD2* gene (rs4880) consists of nucleotide substitution (T, thymine → C, cytosine) and consecutive amino acid substitution of alanine (*Ala*) with valine (*Val*) (*Ala16Val*) which finally will result in decreased efficiency of *SOD2 Val* allele transport in mitochondria by 30–40% and decreased potential in neutralizing superoxide anion [22,23]. Concerning *GPX1* (rs1050450) gene polymorphism, SNP is characterized by nucleotide substitution (C, cytosine → T, thymine), with consecutive substitution of the amino acid proline (*Pro*) with leucine (*Leu*) [24,25]. The *GPX1 C593T* variant is supposed to result in lower enzyme activity and mRNA expression in the presence of *Leu* allele compared with the *Pro* allele [25]. 

There is accumulating evidence connecting the GPX1 and SOD2 genetic variants with cancer development, disease progression, and treatment outcomes in a different types of cancers. Previous reports suggested that SOD expression could be considered a promising prognostic marker for lung, bladder, ovarian, and colon cancer [26]. On the other hand, there is still no general agreement on how is *GPX1 Pro198Leu* gene polymorphism related to cancer risk [27]. Nevertheless, studies which have examined the association of *GPX1* and *SOD2* genetic polymorphisms with bladder cancer risk and aggressiveness are still with conflicting results [28,29,30]. The aim of this study was to investigate the association of *GPX1* (rs1050450) and *SOD2* (rs4880) genetic variants with the risk of UBC development and to evaluate association between these genetic polymorphisms and smoking as known risk factor for UBC. Furthermore, we aimed to determine whether UBC phenotypic features, such as disease stage and pathological grade, were influenced by *GPX1* and *SOD2* genetic polymorphisms. 

## 2. Materials and Methods

### 2.1. Study Design

This prospective single-center hospital-based case-control study included 330 patients with a pathohistologically confirmed diagnosis of UBC who were treated at the Clinic of Urology, University Clinical Center of Serbia, between 1 January 2011 and 1 November 2015. Pathohistological confirmation of UBC, together with assessed pathological stage and grade, was obtained after transurethral resection or cystectomy. A metastatic workup included chest and abdominopelvic CT scan or MRI in selected patients. The control group included 227 age and gender matched subjects whose DNA is a part of a DNA repository of the Institute of Medical and Clinical Biochemistry, Faculty of Medicine, University of Belgrade. None of the participants from the control group had any history of malignant disease. 

All subjects from the study and control group filled out a structured epidemiological questionnaire to estimate demographic and epidemiological data. Informed consent was obtained from all enrolled subjects. The study protocol was approved by the Ethics Committee of the Faculty of Medicine, University of Belgrade (No. 29/XII-14), and the research was carried out in compliance with the Declaration of Helsinki. 

### 2.2. DNA Isolation and Genotyping

A total DNA was purified from leukocytes of 200 µL EDTA-anticoagulated peripheral blood using QIAamp DNA Mini Kit (Qiagen, Inc., Chatsworth, CA, USA), following the manufacturer’s protocol. 

*GPX1* (rs1050450) gene polymorphism was determined by PCR- Restriction Fragment Length Polymorphism (PCR—RFLP) [31]. Components of the PCR reaction mixture (2 μL genomic DNA, 10 μL PCR master mix, 7 μL water), together with primers: forward 5′-GCC GCC GCT TCC AGA CCA T-3′ and reverse 5′-CCC CCC GAG ACA GCA GCA CT-3′ were used to amplify the DNA fragment (128 bp) of the *GPX1* gene. Amplified PCR fragments were digested with Apa1 restriction enzyme (Thermo Fisher Scientific, Waltham, Massachusetts, USA) at 30 °C overnight. The restriction fragments of 128, 67, and 61 bp were visualized on 4% ethidium bromide stained agarose gel after electrophoresis, using ChemiDoc (Bio-Rad Laboratories, Inc., Hercules, CA, USA).

To determine *SOD2* (rs4880) gene polymorphism, the real-time PCR (qPCR) was performed using TaqMan^®^ SNP Genotyping Assays (Life Technologies, Applied Biosystems, Carlsbad, CA, USA, assay ID: C_8709053_10) [31]. The results were visualized using Mastercycler^®^ ep realplex software (Eppendorf, Hamburg, Germany). 

### 2.3. Statistical Analysis

Statistical data analysis was performed using IBM SPSS version 21.0 (IBM, Armonk, NY, USA). Chi-square test was used to analyze the frequency differences between groups, as well as to test the deviation of the genotype distribution from Hardy–Weinberg equilibrium. All categorical variables were presented using frequency (n, %) counts. Continuous variables were compared by Student’s *t*-test, after testing data for normality by Kolmogorov–Smirnov test. Continuous variables were expressed as mean ± standard deviation (SD). Multivariate logistic regression was used to assess the contribution of antioxidant genetic polymorphisms to the risk of UBC. The odds ratio (OR) with 95% confidence interval (CI) was computed after adjusting for gender and age. The level of statistical significance was set at *p* < 0.050.

## 3. Results

Demographic and clinical characteristics of the study group are shown in Table 1. The study group consisted of 330 patients with UBC (average age 65 ± 10.3 years, males n = 230, 70%) and 227 respective controls (average age 63.4 ± 7.9 years, males n = 154, 68%). UBC patients did not differ significantly from the control group in terms of both age and gender (*p* > 0.05). The patient group encompassed a significantly higher number of smokers compared to the control group (75% vs. 49% respectively, *p* < 0.001). 

All patients had a pathohistologically confirmed diagnosis of urothelial bladder carcinoma. As shown in Table 1, the highest number of patients included in this study had the non-metastatic stage of the disease n = 284 (89%), while the metastatic stage of the disease was diagnosed in 33 (11%) patients. Patients with the non-metastatic disease were further divided into two groups according to the pathological tumor stage (pT stage): the group with non-muscle invasive bladder cancer (NMBIC) which consisted of 144 (50.7%) patients (stage pTa n = 10 (4%) and stage pT1 n = 134 (47%)), and the group with muscle-invasive bladder cancer (MIBC) which included 140 (49.3%) patients (stage pT2 n = 91 (32%), stage pT3 n = 26 (9%) and stage pT4 n = 23 (8%)). According to pathohistological grade, urothelial high-grade bladder cancer (HG) was registered in 132 (44.7%), while low grade (LG) was found in n = 115 (39%) cases. Papillary urothelial neoplasm of low malignant potential (PUNLMP) was found in 48 (16.3%) cases.

The distribution of *GPX1* rs1050450 and *SOD2* rs4880 genotypes among UBC patients and controls, as well as their impact on the risk of UBC development, are presented in Table 2. Interestingly, although the carriers of low-activity *GPX1 Leu200Leu* genotype were more frequently found among patients than controls (16.5% vs. 10.3%, respectively) with a slightly increased risk of UBC development in comparison to individuals with referent *GPX1 Pro200Pro* genotype (OR = 1.5, 95%CI = 0.8–2.8, *p* = 0.220), the statistical significance was not reached. However, according to our analysis, only *SOD2* polymorphism reached a statistically significant association with the risk of UBC development. Namely, the risk of UBC was significantly increased among individuals carrying at least one variant *SOD2 Val* allele (*Val16Ala* + *Val16Va*l) compared to the *SOD2 Ala16Ala* homozygotes (OR = 1.55, 95% CI = 1.03–2.3, *p* = 0.030). 

Furthermore, we analyzed the combined effect of *GPX1* rs1050450 and *SOD2* rs4880 genotypes on the risk of UBC development (Table 2). Our results show that the presence of *GPX1 Pro200Pro* genotype in hetero or homozygous carriers of *SOD2 Val allele (Val16Ala + Val16Val)* additionally increased UBC risk. Concisely, individuals carrying referent *GPX1 Pro200Pro* genotype and at least one variant *SOD2 Val* allele (*Val16Ala + Val16Val*) had an over 2 times increased risk of UBC development (OR = 2.16; 95% CI = 1.05–4.42, *p* = 0.036).

Given that smoking is a well-known risk factor for UBC development, the combined effect of smoking and *GPX1* and *SOD2* polymorphisms on the risk for UBC was further analyzed (Table 3). We have found that smoking significantly modifies the risk for UBC development in combination with *SOD2* low-activity genotype. Although smokers homozygous for referent *SOD2 Ala16Ala* genotype had 4.14 fold higher risk of UBC development compared to non-smoking carriers of the same genotype (OR = 4.14, 95% CI = 1.8–9.5, *p* < 0.001), this risk was even 7.5 fold higher in smokers with at least one *SOD2 Val* variant allele (*SOD2 Val16Ala* + *Val16Val*) (OR = 7.5, 95% CI = 3.4–163, *p* < 0.001). However, no added effect of smoking with *GPX1* polymorphism on the risk for UBC was found in our patient population. 

The distribution of *GPX1* and *SOD2* genotypes according to disease stage and pathological grade of UBC is shown in Table 4. We found that the distribution of *GPX1* genotypes was significantly different between patients with NMIBC, MIBC and mUBC (*p* ˂ 0.049), as well as between patients with PUNLMP, low grade and high grade (*p* ˂ 0.018). Our results showed that the frequency of *GPX1 Pro200Pro* genotype was increasing with more advanced disease stage and higher pathological grade (49% for mUBC and 41% *GPX1 Pro200Pro* for high-grade UBC). Moreover, quite the opposite was observed for *GPX1 Leu200Leu* genotype (12% for mUBC and 13% *GPX1 Leu200Leu* for high grade UBC). However, the distribution of *SOD2* genotypes according to disease stage and pathological grade of UBC showed no statistically significant difference in our patient population. 

## 4. Discussion

In this study, we analyzed the association of *GPX1* (rs1050450) and *SOD2* (rs4880) genetic polymorphisms with the risk of UBC development, as well as the combined effect of these genetic polymorphisms and smoking on UBC risk. Our results indicated that the *SOD2* polymorphism is associated with a higher risk of UBC development. Moreover, this risk was even more prominent in smokers with at least one variant *SOD2* allele. On the other hand, considering *GPX1* genetic polymorphism, we have not found an association with the risk of UBC development. However, our results indicated that the *GPX1* polymorphism was associated with the disease aggressiveness.

Our results on the association of *SOD2* polymorphism with the risk of UBC development are comparable with the findings of Hung et al., who showed that the presence of the *SOD2-Val* allele increases the UBC risk by 1.9 times [32] in the Italian population. This is in line with our result since we found that individuals who are carriers of at least one variant *SOD2-Val* allele have about 1.5 times higher probability to develop UBC compared to individuals with the reference *SOD2 Ala16Ala* genotype. On the other hand, the results of a meta-analysis by Cao et al. showed no significant association between *SOD2* polymorphism and the risk of bladder cancer [33]. It is important to note that previously published studies which investigated the association of *SOD2* polymorphism with the development of a different types of cancers also showed conflicting results. In studies conducted on patients with hormone-dependent cancers such as breast, ovarian and prostate cancer, an increased risk was observed in individuals who had the *SOD2-Ala* allele [34,35]. Conversely, in lung, colorectal, and squamous cell carcinoma of the oral cavity, an increased risk was registered in individuals carrying the variant *SOD2-Val* allele [35,36]. 

The interpretation of our findings is consistent with the evidence of the dual role of SOD2 in cancer development [37,38]. Accordingly, as a consequence of the *SOD2* functional polymorphism, decreased enzyme activity leads to a decreased neutralizing capacity of mitochondrial superoxide anion and increase in oxygen free radicals burden. As a result, this significantly disrupts cellular redox homeostasis and may cause genetic and/or epigenetic alterations leading to the dysregulation of oncogenes and tumor suppressor genes that drive the initiation of carcinogenesis [39]. On the other hand, a relative decrease of the amount of hydrogen peroxide due to reduced activity of SOD2 will leave the cell without the stimulus for apoptosis, enabling it to survive and continue with the cascade of transformation into a cancer cell. Several studies have shown that oxidative stress affects signaling pathways associated with cell proliferation [40]. Among them, the epidermal growth factor receptor signaling pathway is especially important, as well as key signaling proteins, such as the nuclear factor erythroid 2-related factor 2 (Nrf2), Ras/Raf, the mitogen-activated protein kinases ERK1/2, and MEK, phosphatidylinositol 3-kinase, phospholipase C, and protein kinase C, which all are redox sensitive [41,42]. Moreover, ROS alters the expression of the p53 suppressor gene which is a key factor in apoptosis. Thus, oxidative stress causes changes in gene expression, cell proliferation and apoptosis, and plays a significant role in tumor initiation and progression [43,44].

Selenium-dependent scavenger of H2O2, GPX1, cooperates between the mitochondria and the cytosol. Hence, a partnership between SOD2 and GPX1 is needed since the mitochondrial resident protein SOD2 converts superoxide to H2O2, which requires further reduction to water by GPX1. Epidemiological and genetic studies referring to the association between these two enzymes suggested that specific polymorphisms in these proteins can interact to impact the risk of cancers [34,45,46,47,48,49]. In our study we found no association regarding the *GPX1* genetic polymorphism and the risk of bladder cancer. Similarly, the study conducted on the Moroccan population showed no association between the *GPX1 Pro198Leu* genetic polymorphism and the risk of bladder cancer [50]. On the other hand, a significant association between *GPX1* genetic polymorphism and the risk of bladder cancer was reported in a few studies [51,52]. In addition, the results of a meta-analysis [33] also showed that carriers of one or both *GPX1-Leu* alleles had an approximately 2-fold higher risk of developing bladder cancer, compared to *GPX1 Pro/Pro* genotype. Besides bladder cancer, during the past two decades, *GPX1 Pro198Leu* polymorphism has been extensively studied in other cancer types [53], mainly breast [54], prostate [55], lung [56], leukemia [57], and colon cancers [58]. However, the association between *GPX1* genetic variants and cancer susceptibility is still controversial and inconclusive probably due to the differences in study cohorts and statistical methods. A comprehensive meta-analysis, including 31 published articles, found that *GPX1 Pro198Leo* polymorphism may promote cancer susceptibility by disturbing the antioxidant balance. They reported that variant *GPX1-Leu* allele carriers (*Pro/Leu* and *Leu/Leu*) have increased cancer risk, especially in Asian subgroups in a dominant genetic model [59]. However, another meta-analysis including 35 published articles suggested no associations between *GPX1 Pro198Leu* polymorphism and cancer risk based on articles with high-quality criteria, whereas strong associations were identified in articles with low-quality criteria [60]. Another possibility for the disagreement of these results may be partially explained by the existing differences in the geographical distribution of *GPX1* genotypes. It is known that the *GPX1-Leu* allele is detected in 36% of whites, 33% of blacks, 5% of Japanese and has not been detected in Chinese so far. This variability in the distribution of *GPX1* genotype should be considered when comparing the results of studies of different ethnic populations [61]. It is noteworthy to mention that when polymorphisms of both *GPX1* and *SOD2* genes were analyzed in combination, the presence of *GPX1 Pro* allele increased the OR for bladder carcinoma risk (1.55 to 2.16 times).

It is widely recognized that smoking is one of the most important risk factors for the development of UBC. Correspondingly, our research confirmed a significant association between smoking and UBC susceptibility. In our study, smokers have about a 4 times higher risk of developing UBC, which is in line with 2–4 times higher risk in smokers reported by other researchers [62,63,64]. Considering that smoking is a significant source of free radicals, we analyzed the combined effect of smoking and polymorphic variants of *GPX1* and *SOD2*. Our results indicated that smokers carrying at least one variant *SOD2-Val* allele (*SOD2 Val16Ala* + *Val16Val* genotype) have a 7.5 times higher risk of developing UBC than nonsmokers carriers of referent *SOD2 Ala16Ala* genotype. This finding is very similar to the results of Hung R et al. who showed around 7 times increased UBC risk in smokers with the variant *SOD2-Val* allele [32]. The relationship between *SOD2* polymorphism and smoking was also reported by Terry and colleagues [65]. Moreover, similar results were obtained in previously published studies in patients with cancers of other locations. Namely, Aynali et al. showed the existence of an increased risk of laryngeal cancer in the population of smokers who had the *SOD2 Val16Val* genotype, but not in smokers with polymorphic variants of *GPX1* [66]. The existence of a significantly higher risk in smokers carrying one or both variant *SOD2-Val* alleles was also confirmed in a study of patients with squamous cell carcinoma of the oral cavity [36]. The results obtained in our study confirm the importance of SOD2 in the reduction of smoking-induced oxidative stress. 

Another goal of our research was to examine the association of genetic polymorphisms of *GPX1* and *SOD2* with the phenotypic characteristics of UBC. Concerning *GPX1* polymorphism, our results indicated that *GPX1* genetic variants were significantly associated with the disease stage and tumor grade. Namely, the *GPX1 Pro198Leu* and *GPX1 Leu198Leu* genotypes, which functionally have a lower activity compared to the *GPX1 Pro198Pro* genotype, were significantly more often detected in patients with a lower disease stage and a lower tumor grade. However, we did not find a significant association between the *SOD2* polymorphism and the UBC stage and grade. By reviewing the literature data, so far only a few studies have been investigating the association of the *GPX1* genetic polymorphism with the UBC disease stage and tumor grade and the reported results differ from those obtained in our study. Namely, a study conducted in Turkey showed that the *GPX1 Leu/Leu* genotype was significantly more often associated with a higher disease stage [51]. In addition, an ethnic study conducted on the Moroccan population [50] showed that the *GPX1 Pro198Leu* polymorphism was not associated with either the disease stage or tumor grade. However, our results are in agreement with the results obtained in a study conducted on patients with breast cancer, showing a protective effect of the *GPX1-Leu* allele [61]. Nonetheless, the interpretation of our results is consistent with the evidence of an anti-angiogenic effect of GPX1 in the processes of differentiation, proliferation and angiogenesis [67]. Specifically, it has been shown that the increased activity of GPX1 in cancer cells leads to a decrease of the intracellular level of hydrogen peroxide. Thereby, with losing the stimulus for apoptosis, the sublethal concentration of hydrogen peroxide enables the survival of the transformed cell. In addition, simultaneous activation of molecular signaling pathways involved in the regulation of differentiation will provide the cell with long enough time to undergo a cascade of changes which lead to the progression of disease stage and the progression of tumor grade. Accordingly, the association of the GPX1 genetic polymorphism with the disease stage and tumor grade which we found in our study indicates that GPX1 could be related to the progression of the disease. Though, when interpreting these results, other molecular genetic factors and mechanisms involved in the context of the phenotypic expression of UBC should also be taken into account. 

The limitation of our study includes first the relatively small number of patients. This may be explained by single-center recruitment. Second, only two genetic polymorphisms were genotyped. Since the number of causal SNPs, gene-gene and gene-gene interactions with environmental factors are still unknown, the single gene based approach in genetic studies may have limited value. Urothelial bladder cancer is a multifactorial disease, meaning that complex interactions between genetic and environmental factors are considered to have an impact on the onset and progression of the disease, as well as treatment outcomes. 

## 5. Conclusions

In conclusion, we found that genetic polymorphism of the SOD2 antioxidant enzyme is associated with the risk of UBC development independently and in combination with cigarette smoking. Furthermore, we found that genetic polymorphism of the GPX1 antioxidant enzyme is associated with the phenotypic characteristics of UBC such as disease stage and tumor grade, i.e., with the aggressiveness of the disease. Further research based on the examination of different gene-gene and gene-environmental interactions between several different genetic polymorphisms and environmental factors, in the future will clarify the picture of complex molecular events that exist in patients with urothelial bladder cancer.

## Figures and Tables

**Table 1:** Demographic and clinical characteristics of patients with UBC and the controls.

Characteristic	Patients	Controls	OR (95% CI)	*p*
Gender, *n* (%)				
Female	100 (30)	73 (32)	1.0	
Male	230 (70)	154 (68)	0.8 (0.54–1.21)	0.300
Age, mean ± SD, y	65 ± 10.3	63.4 ± 7.9		0.050
Smoking, *n* (%)				
Non Smokers	80 (25)	112 (51)	1.0	
Smokers	247 (75)	108 (49)	3.9 (2.6–6)	<0.001
Disease stage, *n* (%)				
Nonmetastatic	284			
NMIBC	144 (50.7)			
pTa	10 (4%)			
pT1	134 (47%)			
MIBC	140 (49.3)			
pT2	91 (32%)			
pT3	26 (9%)			
pT4	23 (8%)			
*Metastatic*	33			
Pathological grade (WHO 2004), n (%)				
PUNLMP	48 (16.3)			
Low Grade (LG)	115 (39)			
High Grade (HG)	132 (44.7)			

OR, odds ratio; CI, confidence interval; NMIBC, Non-muscle invasive Bladder Cancer (NMIBC); MIBC, Muscle invasive bladder cancer; WHO, World Health Organization; PUNLMP—Papillary urothelial neoplasm of low malignant potential.

**Table 2:** The association of GPX1 and SOD2 polymorphisms with the risk of UBC development.

Genotype	Patients n, %	Controls n, %	OR (95% CI)	*p*
*GPX1 rs1050450*				
*Pro200Pro*	104 (33.0)	67 (38.5)	1.0 ^a^	
*Pro200Leu*	159 (50.5)	89 (51.1)	1.07 (0.7–1.6)	0.750
*Leu200Leu*	52 (16.5)	18 (10.3)	1.5 (0.8–2.8)	0.220
*Pro200Leu + Leu200Leu*	211 (67.0)	107 (61.5)	1.15 (0.8–1.7)	0.500
*SOD2 rs4880*				
*Ala16Ala*	65 (23)	66 (32)	1.0^a^	
*Val16Ala*	155 (56)	99 (46)	1.65 (1.07–2.53)	0.020
*Val16Val*	60 (21)	47 (22)	1.33 (0.79–2.23)	0.290
*Val16Ala + Val16Val*	215 (77)	146 (69)	1.55 (1.03–2.3)	0.030
*GPX1/SOD2*				
*Pro200Pro/Ala16Ala*	22 (8)	24 (15)	1.0 ^a^	
*Pro200Leu + Leu200Leu/Ala16Ala*	43 (16)	29 (17)	1.55 (0.72–3.33)	0.260
*Pro200Pro/Val16Ala + Val16Val*	73 (26)	40 (24)	2.16 (1.05–4.42)	0.036
*Pro200Leu + Leu200Leu/Val16Ala + Val16Val*	139 (50)	73 (44)	1.96 (1.01–3.78)	0.047

OR, odds ratio adjusted for gender and age; CI, confidence interval; ^a^ reference group.

**Table 3:** Combined effect of smoking and GPX1 and SOD2 genotypes on the risk of UBC.

Genotype	Patients, n, %	Controls n, %	OR (95% CI)	*p*
*GPX1 rs1050450*				
*Pro200Pro/nonsmoker*	23 (7)	28 (16)	1.0 ^a^	
*Pro200Pro/smoker*	80 (25)	37 (21)	3.33 (1.6–6.0)	<0.001
*Pro200Leu + Leu200Leu/nonsmoker*	55 (18)	49 (28)	1.32 (0.6–2.7)	0.441
*Pro200Leu + Leu200Leu/smoker*	159 (50)	62 (35)	3.67 (1.8–7.2)	<0.001
*SOD2 rs4880*				
*Ala16Ala/nonsmoker*	11 (4)	28 (13)	1.0 ^a^	
*Ala16Ala/smoker*	53 (19)	38 (18)	4.14 (1.8–9.5)	<0.001
*Val16Ala + Val16Val/nonsmoker*	58 (21)	78 (38)	1.9 (0.9–4.2)	0.100
*Val16Ala + Val16Val/smoker*	155 (56)	63 (30)	7.5 (3.4–16.3)	<0.001

OR, odds ratio adjusted for gender and age; CI, confidence interval; ^a^ reference group.

**Table 4:** Distribution of GPX1 and SOD2 genotypes according to disease stage and pathological grade of UBC.

Characteristic	Genotype *GPX1 rs1050450*				
	Patients, n	*Pro200Pro* n (%)	*Pro200Leu* n (%)	*Leu200Leu* n (%)	*p*
Disease stage, n (%)					
NMIBC	138	38 (27)	73 (53)	27 (20)	
MIBC	137	57 (42)	58 (42)	22 (16)	0.049
mUBC	33	16 (49)	13 (39)	4 (12)	
Pathological grade, n (%)					
PUNLMP	44	7 (16)	29 (66)	8 (18)	
Low Grade (LG)	112	39 (35)	49 (44)	24 (21)	
High Grade (HG)	129	53 (41)	59 (46)	17 (13)	0.018
	Genotype *SOD2 rs4880*				
	Patients, n	*Val16Val* n (%)	*Val16Ala* n (%)	*Ala16Ala* n (%)	*p*
Disease stage, n (%)					
NMIBC	120	23 (19)	65 (54)	32 (27)	
MIBC	126	30 (24)	70 (56)	26 (20)	0.453
mUBC	33	9 (27)	17 (52)	7 (21)	
Pathological grade, n (%)					
PUNLMP	30	6 (20)	16 (53)	8 (27)	
Low Grade (LG)	102	21 (21)	58 (57)	24 (22)	
High Grade (HG)	122	29 (24)	65 (53)	28 (23)	0.960

NMIBC, Non-muscle invasive Bladder Cancer (NMIBC); MIBC, Muscle invasive bladder cancer; WHO, World Health Organization; mUBC, metastatic urothelial bladder carcinoma; PUNLMP—Papillary urothelial neoplasm of low malignant potential.

## Data Availability

The data supporting the reported results can be provided upon request in the form of datasets available at the Clinic of Urology, University Clinical Centre of Serbia and the Institute of Medical and Clinical Biochemistry, Faculty of Medicine, University of Belgrade.
